# CASK Silence Overcomes Sorafenib Resistance of Hepatocellular Carcinoma Through Activating Apoptosis and Autophagic Cell Death

**DOI:** 10.3389/fonc.2021.681683

**Published:** 2021-06-23

**Authors:** Bisha Ding, Chang Bao, Luqi Jin, Liang Xu, Weimin Fan, Weiyang Lou

**Affiliations:** ^1^ Department of Breast Surgery, The First Affiliated Hospital, College of Medicine, Zhejiang University, Hangzhou, China; ^2^ Program of Innovative Cancer Therapeutics, Division of Hepatobiliary and Pancreatic Surgery, Department of Surgery, First Affiliated Hospital, College of Medicine, Key Laboratory of Combined Multi-Organ Transplantation, Ministry of Public Health, Key Laboratory of Organ Transplantation, Zhejiang University, Hangzhou, China; ^3^ Department of Cell Biology, Zhejiang University School of Medicine, Hangzhou, China

**Keywords:** autophagy, apoptosis, HCC, CASK, sorafenib resistance

## Abstract

Hepatocellular carcinoma (HCC) patients usually fail to be treated because of drug resistance, including sorafenib. In this study, the effects of CASK in HCC were investigated using gain- or loss-of-function strategies by performing cell counting kit-8 assay, colony formation assay, flow cytometry, transmission electron microscopy, immunofluorescent confocal laser microscopy, tumor xenograft experiment and immunohistochemistry staining. The current results suggested that CASK expression was positively associated with sorafenib resistance and poor prognosis of HCC. Moreover, inhibition of CASK increased the role of sorafenib partially by promoting apoptosis and autophagy, while CASK overexpression presented the opposite effects. Besides, when treatment with sorafenib, inhibition of apoptosis using the pan-caspase inhibitor Z-VAD-FMK and inhibition of autophagy using autophagy inhibitor 3-Methyladenine (3-MA) or small interfering RNA (siRNA) of LC3B all significantly reversed CASK knockout-induced effects, suggesting that both apoptosis and autophagy were involved in CASK-mediated above functions and autophagy played a pro-death role in this research. Intriguingly, similar results were observed *in vivo*. In molecular level, CASK knockout activated the c-Jun N-terminal kinase (JNK) pathway, and treatment with JNK inhibitor SP600125 or transiently transfected with siRNA targeting JNK significantly attenuated CASK knockout-mediated autophagic cell death. Collectively, all these results together indicated that CASK might be a promising biomarker and a potential therapeutic target for HCC patients.

## Introduction

Hepatocellular carcinoma, approximately 750,000 new cases occurred per year, is the sixth most frequently neoplasm and ranks the third leading cause of cancer-related deaths worldwide (1–3). Currently, 30% HCC patients present with advanced stages and treatment options for them are limited, partially accounting for the dismal survival rate of HCC (4). The molecular targeted agent, sorafenib, remains one of the first-line systemic drugs for advanced HCC patients (5, 6). However, sorafenib resistance limits its efficacy in HCC. Although some conditions or pathways leading to sorafenib resistance, including AKT activation, hypoxic environment, Epithelial–mesenchymal transition (EMT), cancer stem cells/tumor-initiating cells, Epidermal growth factor receptor (EGFR) activation, c-Jun activation and autophagy have been reported (7), more efforts should be input to further elucidate the complicated sorafenib resistance mechanism and improve the outcome of sorafenib treatment in HCC patients.

Calcium/calmodulin-dependent serine protein kinase (CASK), a scaffold protein from membrane-associated guanylate kinase (MAGUK) protein family, plays a significant role in the neuronal system (8, 9). However, the research about this gene in cancer remains limited. To date, some previous studies demonstrated that CASK closely links to tumor development. For example, Wei et al. found that high expression level of CASK was associated with poor prognosis and progression of colorectal cancer (10); Zhou et al. revealed that decreased the expression of miR-203 promoted the proliferation and invasion of gastric cancer through targeting CASK (11). These reports suggest that CASK may play an important role in cancer, while the research of CASK in HCC is poorly explored but necessary to be further studied.

Apoptosis, or programmed cell death, is a common mechanism sensitizing cancer cells to chemotherapy agents, and is regulated by lots of apoptosis-related proteins and pathways (12). Besides, autophagy is a self-degradative cellular process, and it plays a key role in the development and progression of tumor, including drug resistance (13). Chemotherapy agents, such as sorafenib and fluorouracil (5-FU), may induce autophagy, but the role of autophagy is indistinct. On the one hand, autophagy can lead to chemoresistance through a cell survival mechanism (14–16). On the other hand, overactivated autophagy can lead to autophagic cell death or non-apoptotic form of programmed cell death and relieve drug resistance (17–21). Interestingly, several lines of evidence show that apoptosis and autophagy closely cross talk with each other (22, 23). Therefore, targeting apoptosis or autophagy may provide a potential and promising therapeutic strategy to overcome chemoresistance and relieve progression for patients with cancer.

Our data suggested that high expression of CASK was positivity associated with sorafenib resistance of HCC cells and poor prognosis of HCC patients. Furthermore, we confirmed that depletion of CASK augmented the chemosensitivity of HCC by activating apoptosis and enhanced JNK/c-Jun pathway-mediated autophagic cell death. Overall, our study demonstrate that CASK might be a potential biomarker for HCC patients and provides a brand-new strategy for HCC therapy, and it is worthful for in-depth study in the further.

## Material and Methods

### Clinical Samples

Some 60 HCC tissues and paired normal tissues were obtained from the First Affiliated Hospital of Zhejiang University (diagnosed from 2017 to 2018). All patients signed the informed consent. This study was approved by the Medical Ethics Committee of the First Affiliated Hospital of Zhejiang University. The diagnosis of patient was confirmed by histopathological examination.

### Cell Culture and siRNA Transfection

In this study, SMMC-7721 and SK-Hep-1 cells were purchased from the Cell Bank of the Chinese Scientific Academy (Shanghai, China), SMMC-7721-sora cells were purchased from the MEIXUAN Biological Science and Technology Ltd (Shanghai, China). SMMC-7721 cells were cultured in RPMI-1640 medium (Gibco, Life Technologies, Carlsbad, CA, USA) supplemented with 10% fetal bovine serum (FBS; Biological Industries, Cromwell, CT, USA). SK-Hep-1 cells were cultured in Dulbecco’s modified Eagle’s medium (DMEM, Gibco, Life technologies, Carlsbad, CA, USA) containing 100 units/ml streptomycin and penicillin with 10% FBS. SMMC-7721-sora cells were cultured in RPMI-1640 medium (Gibco, Life Technologies, Carlsbad, CA, USA) supplemented with 10% fetal bovine serum (FBS; Biological Industries, Cromwell, CT, USA) and 2,000 ng/ml sorafenib. All cell lines were maintained at 37°C and 5% CO_2_ in incubator.

siRNAs for CASK, LC3B and JNK were synthesized by RiboBio Co., Ltd (Guangzhou, China) (**Table S1**). HCC cells were seeded in 6- or 96-well plates, transfected after 12 h according to the manufacturer’s protocol and treated with drug for 72 h after transfection.

### Plasmid Construction and Transfection

CASK expression was knocked out using CASK sgRNA CRISPR/Cas9 system (target sequences: 5-CGACGACGACGTGCTGTTCG-3) and stable expression cell lines were selected using puromycin. pcDNA3.1 and pcDNA-CASK plasmids were designed by Repbio Co., Ltd (Hangzhou, China) and stable expression cell lines were selected using G418. All these transfections were performed according to the manufacturer’s instruction.

### Cell Viability Assay and Colony Formation Assay

Cell viability assays were performed by the CCK8 assays. Cells were plated into 96-well plates with 3,000 cells per well. After 12 h, cells were transfected. Next, cells were exposed to drug treatment and cultured for 72 h. Subsequently, 10 ul of CCK8 was added to each well for 4 h, then the OD value was measured at 450 nm. HCC cells were seeded into 6-well plates for colony formation assay (1,000 cells/well for SMMC-7721/SMMC-7721-sora; 350 cells/well for SK-Hep-1). After being maintained at 37°C and 5% CO_2_ in incubator for 2 weeks, cells were stained with Wright–Giemsa stain according to the manufacturer’s instructions. The number of colonies more than 50 cells/colony were counted.

### Flow Cytometry Analysis

The cells were divided into different groups according to the experimental requirements (si-NC, si-CASK-3, si-NC + sorafenib, si-CASK-3 + sorafenib; pcDNA 3.1 + sorafenib, pcDNA-CASK + sorafenib). At first, 20 × 10^4^ si-NC/si-CASK-3 or pcDNA 3.1/pcDNA-CASK transfected cells were plated into 6-well plates, then cultured with or without sorafenib for 72 h according to experiment groups. An Annexin V-FITC/PI apoptosis kit (Multi Sciences, Biotech, China) was used to detect cell apoptosis based on the manufacturer’s protocol. Data analyses were performed with BD FACSCalibur™ flow cytometry system.

### RNA Extraction and Quantitative Real-Time PCR (qRT-PCR)

Total RNA was extracted from tissues and cells using RNAiso plus Reagent (TaKaRa, Kusatsu, Japan) and reverse transcribed to cDNA using PrimeScript RT Reagent Kit (TaKaRa, RR0037A). cDNA was analyzed by qRT-PCR using SYBR Premix Ex Taq (TaKaRa, RR420A). GAPDH was used as an endogenous control. The relative expression values were calculated using 2^−ΔΔCT^ method. Gene-specific primers were listed in **Table S1**.

### Chemotherapeutic Agents and Antibodies

The antibodies including Anti-GAPDH, Anti-CASK, Caspase-7, Anti-LC3B, SQSTM1/p62, Beclin-1, Anti-JNK1+JNK2+JNK3, Anti-phospho-JNK, Anti-c-Jun, Anti-phospho-c-Jun, Anti-MRP3, Anti-ABCG2 were listed in **Table S2**. JNK inhibitor SP600125 (MB5595), 3-Methyladen (3-MA, MB5063), Z-VAD-FMK (MB3313), sorafenib (MB1666), doxorubicin (MB1087), daunorubicin (MB1074), decitabine (5-Aza, MB1075), G418 (MB1733), puromycin (MB2005) were purchased from Dalian Meilun Biotechnology.

### Western Blot Analysis

Total proteins were lysed with RIPA buffer containing with protease inhibitor and phosphatase inhibitor on ice and centrifuged at 14,000 rpm to remove the debris. A BCA protein assay kit (Beyotime Biotec, China) was used to measure concentrations of proteins. Equal amount of protein sample was subjected to SDS-polyacrylamide gel electrophoresis (SDS-PAGE) and transferred to PVDF membrane. The membrane was blocked with 5% non-fat milk at room temperature for 1 h, and incubated with primary antibodies overnight at 4°C. After that, the membrane was incubated with secondary antibody for 1 h at room temperature. The results were visualized and analyzed by ECL detection solution (Thermo Scientific™) and Image Lab software (Bio-Rad), respectively.

### Transmission Electron Microscopy (TEM)

About 5 × 10^6^ si-NC or si-CASK-3 transfected cells treated with or without 12.5 μM sorafenib were collected and fixed in 2.5% glutaraldehyde at least for 4 h. All the samples were treated with 1% osmium tetroxide and dehydrated in graded concentrations of ethanol and acetone, finally samples were embedded in Durcupan resin. Ultrathin sections (70 nm) were examined under a JEM-1230 electron microscope (JEOL, Japan) at 80 kV.

### Tumor Xenograft Experiments

All the experiments were approved by the Institutional Animal Care and Use Committee of Zhejiang University. Male nude athymic mice (3–5 weeks old) were purchased from SLAC Laboratory Animal CO., Ltd (Shanghai, China). A stable CASK knockout cell line of SMMC-7721-sora was established (SMMC-7721-sora sg-CASK). In order to determine the effect of CASK knockout on sorafenib resistance *in vivo*, a total of 5 × 10^6^ cells were injected into the right flank of each immune-deficient nude mice (control group or sg-CASK group, N = 10 per group). About 6 days later, animals in each group were divided into another two groups (Dimethyl sulfoxide (DMSO) treated or sorafenib treated, 10 mg/kg) and administered every 3 days. Additionally, to explore the role of CASK regulates autophagy and augments sorafenib sensitivity *in vivo*, a total of 5 × 10^6^ cells were injected into the immune-deficient nude mice. Some 6 days later, all of the animals were divided into four groups (no treated or 3-MA treated or sorafenib treated or combination of 3-MA and sorafenib treated, 3-MA: 20 mg/kg, sorafenib: 10 mg/kg, N = 4 per group) and administered every 3 days. Mice body weight and tumor volume (1/2 × length × width^2^) were measured every 3 days, continue to day 24. Besides, after the animals were terminated, tumor tissues were separated and weighted.

### Immunofluorescent Confocal Laser Microscopy

SMMC-7721-sora control cells or SMMC-7721-sora sg-CASK cells were grown on coverslips and transfected with a GFP-LC3 plasmid overnight and pretreated with or without 20 μM SP600125 for 2 h. Then, cells were treated with 12.5 μM sorafenib or not treated for another 36 h. After that, cells were fixed with 4% paraformaldehyde for 15 min and stained with 4′,6-diamidino-2-phenylindole (DAPI) for 5 min. Images were taken under confocal fluorescence microscopy (Nikon AIR confocal microscope).

### Immunohistochemistry (IHC) Staining

Hematoxylin and eosin (H&E) staining and immunohistochemistry (IHC) staining were performed to gain the expression of CASK, LC3B, p-JNK and p-c-Jun in 4-μM-thick paraffin-embedded sections from tumor xenografts. Slides were examined using an optical microscope (Olympus). At least seven randomly selected 40× fields of Ki67 staining were visualized and the percentage of positive nuclei was quantified using Image J software (NIH, Bethesda, USA).

### Statistics

Experimental data were performed using GraphPad Prism software (version 7.0.3) and expressed as mean ± standard deviation (SD) of at least three independent experiments. Differences between two groups were analyzed using unpaired Student’s *t*-test and p value less than 0.05 was indicated statistically significant.

## Results

### Hypomethylation-Associated Upregulation of CASK Expression in HCC Was Positively Correlated With Poor Prognosis

**Figure 1:**
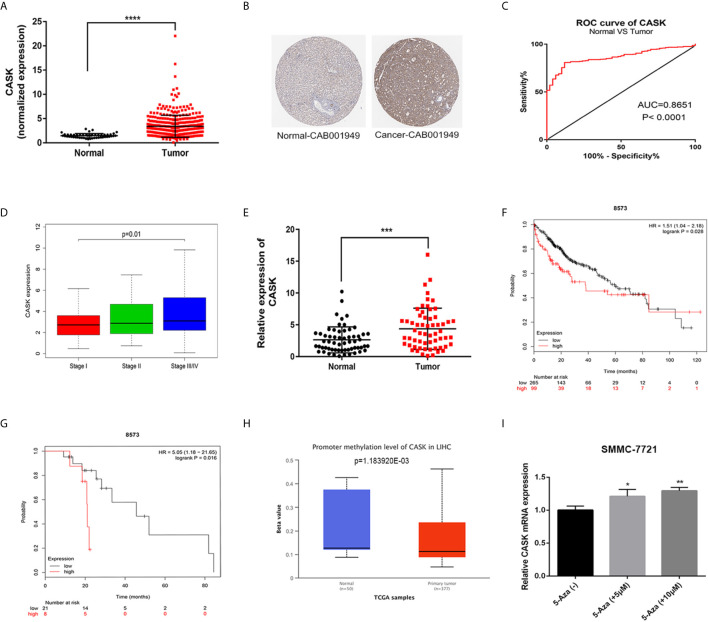
CASK expression was elevated in HCC, and correlated with sorafenib resistance and poor prognosis. **(A)** Expression of CASK in HCC tissues were compared with normal tissues from TCGA database. **(B)** IHC of CASK expression in HCC tissue and normal tissue through using HPA database. **(C)** ROC curve analysis of CASK in HCC. **(D)** The correlation analysis between CASK levels and the stage in HCC cancer patients. **(E)** qRT-PCR analysis of CASK mRNA expression levels in 60 paired HCC tissues and corresponding normal tissues. **(F)** The Kaplan–Meier survival analysis of overall survival for HCC patients. **(G)** The Kaplan–Meier survival analysis of overall survival for HCC patients with sorafenib treated. **(H)** The promoter region methylation levels of CASK in HCC and normal tissues from UALCAN database (http://ualcan.path.uab.edu/). **(I)** qRT-PCR analysis detecting the CASK levels in SMMC-7721 cells after treatment with 5 μM 5-azacytidine (5-Aza) or 10 μM 5-azacytidine (5-Aza) for 72 h. *P < 0.05, **P < 0.01, ***P < 0.001, ****P < 0.0001.

To explore the potential function of CASK in HCC, we first analyzed the expression of CASK through bioinformatic analysis and experimental validation. The results showed that the messenger RNA (mRNA) expression of CASK was significantly upregulated in HCC tissues compared with normal liver tissues in The Cancer Genome Atlas (TCGA) (https://cancergenome.nih.gov/) (**Figure 1A**), and IHC staining performed by the human protein atlas (HPA) database (https://www.proteinatlas.org/) showed strongly positive CASK staining in HCC cancer tissue, but weakly positive staining in normal tissue (**Figure 1B**). Receiver operating characteristic (ROC) curve analysis indicated that the expression of CASK could effectively distinguish HCC from normal tissues (**Figure 1C**). And upregulation of CASK expression was associated with advanced stage in HCC (**Figure 1D**). Furthermore, we compared the expression of CASK in 60 paired HCC samples and corresponding normal samples and found that CASK was markedly upregulated in HCC cancer samples (**Figure 1E**). Besides, the Kaplan–Meier analysis (http://kmplot.com/analysis/) indicated that HCC patients with high-CASK expression demonstrated poor overall survival rate, and consistent result was observed in HCC patients treated with sorafenib (**Figures 1F, G**). Promoter DNA methylation is closely related with gene expression (24, 25). Thus, a possible link between promoter hypomethylation and CASK high expression in HCC was investigated. According to the analysis from UALCAN database (http://ualcan.path.uab.edu/), we found that promoter methylation level of CASK was remarkably decreased in HCC tumor tissues compared with normal tissues (**Figure 1H**). Moreover, SMMC-7721 cells treated with 5 or 10 μM demethylation agent, 5-AZA, significantly increased CASK mRNA levels (**Figure 1I**). Collectively, all the data suggested that promoter hypomethylation-associated CASK upregulation in human HCC was positively related with sorafenib resistance and poor prognosis.

### Knockdown of CASK Inhibited HCC Proliferation, Promoted HCC Apoptosis and Enhanced the Sorafenib Sensitivity *In Vitro*

**Figure 2:**
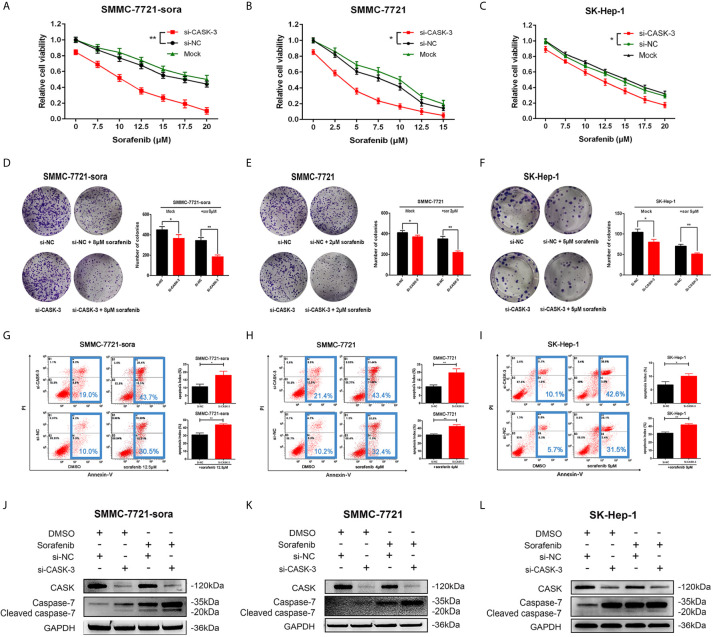
CASK downregulation inhibited HCC cell growth, promoted HCC cell apoptosis and attenuated sorafenib resistance. **(A–C)** CCK-8 assay analysis of the impact of CASK knockdown combines with various concentrations of sorafenib on SMMC-7721-sora, SMMC-7721 and SK-Hep-1 cells growth. **(D–F)** Colony information assay showing the effects of CASK knockdown on SMMC-7721-sora, SMMC-7721 and SK-Hep-1 cells growth with or without sorafenib treated. **(G–I)** The apoptosis rate in CASK knockdown or control cells with or without sorafenib treated was measured by flow cytometry analysis. **(J–L)** Western blotting analysis of apoptosis-related protein levels in CASK knockdown or control cells, with or without sorafenib treated (sorafenib treated concentration: SMMC-7721: 10 μM, SMMC-7721-sora: 20 μM, SK-Hep-1: 15 μM; treated time: 72 h). *P < 0.05, **P < 0.01.

To explore the role of CASK in HCC, we detected the mRNA expression levels of CASK in 3 HCC cell lines (SMMC-7721, SMMC-7721-sora and SK-Hep-1) and 1 normal liver cell line (QSG7701) firstly. The results showed that the expression level of CASK was significantly higher in HCC cancer cell lines than in normal cell line (**Figure S1A**). Quite interestingly, we found that HCC cell lines with lower levels of CASK were more sensitive to sorafenib treatment (The IC50 to sorafenib of SMMC-7721 cell was 9.97 μM, SK-Hep-1 cell was 15 μM and SMMC-7721-sora cell was 22.2 μM). In addition, the presence of sorafenib affected the expression of CASK in SMMC-7721 and SMMC-7721-sora cells in a dose-dependent manner (**Figures S1B–C**). Next, SMMC-7721-sora, SMMC-7721 and SK-Hep-1 cells were transfected with specific siRNAs targeting CASK (si-CASK-1, si-CASK-2, si-CASK-3) and negative control si-NC, the qRT-PCR assay indicated that si-CASK-3 with the best effect in inhibiting CASK expression (**Figures S1D–F**). Additionally, the western blotting analyses were further performed to verify the effect of si-CASK-3 in SMMC-7721-sora, SMMC-7721 and SK-Hep-1 cells (**Figure S1G**). And CCK8 assay showed that after decreasing expression of CASK, the sorafenib treatment was significantly sensitive in various concentrations in SMMC-7721-sora, SMMC-7721 and SK-Hep-1 cells (**Figures 2A–C**). Besides, overexpression of CASK showed opposite consequences in SMMC-7721-sora, SMMC-7721 and SK-Hep-1 cells (**Figures S2A, B**). For colony information assay, HCC cells were treated with si-CASK-3 or si-NC for 12 h, then cultured with sorafenib (sorafenib concentrations: 8 μM for SMMC-7721-sora, 2 μM for SMMC-7721, 5 μM for SK-Hep-1) for 14 days. The assay further indicated that downregulation of CASK significantly decreased clonogenicity of SMMC-7721-sora, SMMC-7721 and SK-Hep-1 cells, and the effect was more pronounced when co-treated with sorafenib (**Figures 2D–F**). Furthermore, CASK upregulation combined with sorafenib treated notably increased clonogenicity of SMMC-7721-sora, SMMC-7721 cells and SK-Hep-1 cells compared with control groups (**Figures S2C, D**). To further define the mechanism of CASK-induced sorafenib chemoresistance, cells transfected with si-CASK-3 or si-NC cultured with varying concentrations of sorafenib or DMSO were analyzed for the apoptotic marker changes by the flow cytometric analyses. Markedly, a significant increase in apoptosis was observed in CASK-knockdown cells compared with control cells, and the effect was more noticeable when combined with sorafenib (**Figures 2G–I**). To further confirm these consequences, cleaved caspase-7, one marker of apoptosis, was monitored by western blotting analysis. As expected, the level of cleaved caspase-7 was increased upon inhibition of CASK, with or without sorafenib treatment (**Figures 2J–L**). In contrast, overexpression of CASK in SMMC-7721-sora, SMMC-7721 and SK-Hep-1 cells had the opposite effects, leading to inhibit sorafenib-induced apoptosis (**Figures S2E, F**).

### Silence of CASK Increased Autophagy of HCC Cells and Activated the JNK/c-Jun Signaling Pathway

**Figure 3:**
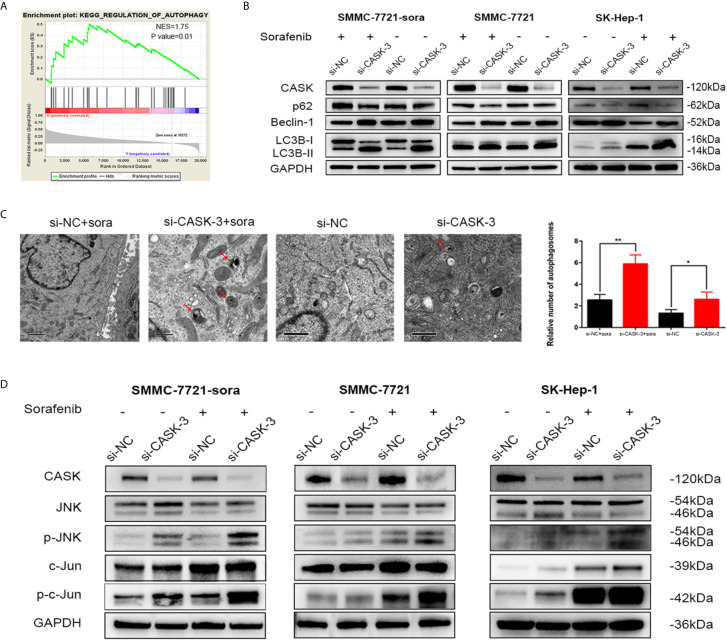
CASK depletion increased autophagy and activated JNK/c-Jun signaling pathway. **(A)** Enrichment plots from Gene Set Enrichment Analysis (GSEA). The GSEA results indicated that the regulation of autophagy was differentially enriched in HCC with high CASK expression. (NES, normalized enrichment score) **(B)** Western blot analysis showing levels of autophagy-related proteins in CASK knockdown cells with the presence or absence of sorafenib (treated concentration: IC50 of sorafenib; treated time: 72 h) **(C)** Transmission electron microscopy (TEM) shows the number of autophagosomes were evaluated in CASK knockdown cells compared with control cells. **(D)** Western blotting analysis of the impact of CASK knockdown on the activity of JNK and c-Jun in SMMC-7721-sora, SMMC-7721 and SK-Hep-1 cells treated with or without sorafenib (sorafenib treated concentration: SMMC-7721: 10 μM, SMMC-7721-sora: 20 μM, SK-Hep-1: 15 μM; treated time: 72 h). *P < 0.05, **P < 0.01.

In order to further discover the potential molecular mechanism underlying the increased drug sensitivity induced by decreased CASK expression, we performed the gene set enrichment analysis (GSEA) using the data from TCGA. We interestingly found that CASK expression was closely related with regulation of autophagy (**Figure 3A**). Since autophagy is a significant regulatory progress in maintaining the cellular homeostasis, and plays a dual role in resistance, we hypothesized that regulation of autophagy may participated in CASK-mediated sorafenib resistance. To test this, we investigated the levels of LC3B, p62 and Beclin-1 in SMMC-7721-sora, SMMC-7721 and SK-Hep-1 cells. Western blotting results showed that CASK knockdown significantly increased the expression of autophagic marker LC3B-II and Beclin-1, and decreased SQSTM1/p62 expression, with or without sorafenib treatment, whereas CASK overexpression presented opposite results when treated with sorafenib (**Figure 3B** and **Figure S3A**). The TEM results revealed that CASK-knockdown cells contained more autophagosomes in the cytoplasm compared with control cells, with or without sorafenib treated (**Figure 3C**).

Increasing studies have confirmed that JNK pathway activation plays a pivotal role in regulating autophagy, and is closely related with chemoresistance and tumor progression (26–28). To further investigate the mechanism of CASK knockdown-induced autophagy, the effect of CASK knockdown on the JNK pathway was detected. Western blotting analysis indicated that CASK knockout indeed led to the increase of the protein levels of phosphorylated-JNK (p-JNK) and phosphorylated-c-Jun (p-c-Jun) in SMMC-7721-sora, SMMC-7721 and SK-Hep-1 cells with or without sorafenib treated (**Figure 3D**).

### Knockout of CASK Inhibited HCC Cell Tumorigenesis and Increased the Effect of Sorafenib *In Vivo*

**Figure 4:**
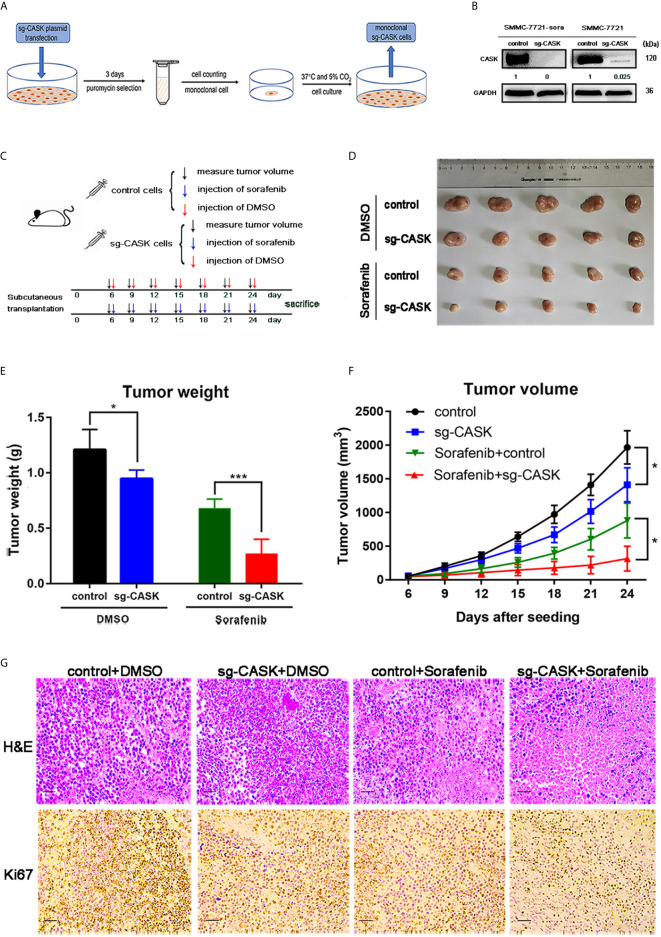
CASK knockout suppressed tumorigenesis and sorafenib resistance of HCC *in vivo*. **(A)** The flow chart for screening stable CASK knockout cell lines using CRISPR Cas9. **(B)** The western blot analysis indicates the protein expression of CASK in SMMC-7721-sora and SMMC-7721 cell lines stably-transfected by sgRNA against CASK using CRISPR Cas9. **(C)** Overall workflow of *in vivo* experiments. **(D–F)** The subcutaneous tumor models were built by SMMC-7721-sora with stable CASK knockout cells or control cells with following treatment of DMSO or sorafenib. The tumor weights and tumor volumes were measured and quantified. **(G)** Representative images of tumor samples with H&E and Ki67 staining. Scale bar: 100 μM. *P < 0.05, ***P < 0.001.

SMMC-7721-sora and SMMC-7721 cells with stable knockout of CASK using CRISPR/Cas9 were screened out for the next research, and western blotting assay was performed to validate the effective knockout of CASK (**Figures 4A, B**). Then, SMMC-7721-sora (control cells) and SMMC-7721-sora sg-CASK cells were selected to conduct *in vivo* analysis. As shown in the diagram (**Figure 4C**), a total of 5 × 10^6^ control cells or sg-CASK cells in 100 ul PBS were injected into the mice, respectively. Some 6 days later, each group mice were further injected with 10 mg/kg of sorafenib every 3 days or DMSO every 3 days, and tumor volumes were measured. We found that the tumors derived from control cells grew evidently faster than those from sg-CASK cells, and the difference was more obvious when treated with sorafenib (**Figure 4D**). Likewise, tumor weight of xenografts derived from CASK suppression demonstrated a superior response to sorafenib compared to controls (**Figure 4E**). Consistently, the mean volume of tumors in CASK knockout groups showed markedly smaller than in controls groups, especially in combination of sorafenib treated (**Figure 4F**). H&E staining and Ki67 staining further indicated that knockout of CASK significantly inhibited proliferation (**Figure 4G**). Together, these results suggested that depletion of CASK inhibited HCC cell tumorigenesis, increased apoptosis and enhanced the therapeutic effect of sorafenib *in vivo*.

### CASK Depletion Modulated Autophagic Cell Death-Mediated Sorafenib Sensitization Through Activating JNK/c-Jun Signaling Pathway

**Figure 5:**
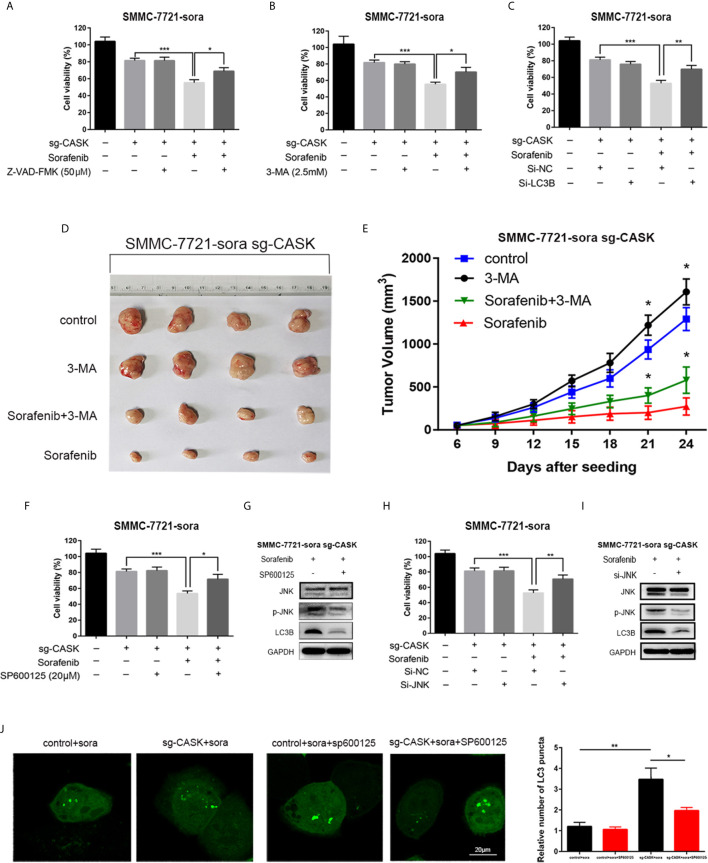
CASK depletion increased autophagic cell death through JNK/c-Jun signaling pathway. **(A)** Z-VAD-FMK partly attenuates CASK knockout-mediated sorafenib-induced HCC cell death by CCK8 assay. SMMC-7721-sora cell was pretreated with or without Z-VAD-FMK (50 μM) for 2 h followed by exposure to 10 μM sorafenib for 24 h. **(B)** 3-MA partly attenuates CASK knockout-mediated sorafenib-induced SMMC-7721-sora cell death by CCK8 assay. Cells pretreated with or without 3-MA (2.5 mM) for 2 h, and following treated with 10 μM sorafenib for another 24 h. **(C)** si-LC3B partly reversed CASK knockout combine with sorafenib treatment-induced cell death by CCK8 assay. Cells pretreated with or without transiently transfected with si-LC3B for 12 h, and incubated with 10 μM sorafenib for another 72 h. **(D)** Representative image of CASK knockout xenograft tumors. **(E)** Tumor volume in each group. **(F)** SP600125 partly decreased CASK knockout-mediated sorafenib-induced SMMC-7721-sora cell death by CCK8 assay. Cells pretreated with or without 20 μM SP600125 for 2 h, and following treated with 10 μM sorafenib for another 24 h. **(G)** Western blotting were performed to detect the expression of p-JNK and LC3B following the treatment of SP600125 in SMMC-7721-sora sg-CASK cell. **(H)** si-JNK partly decreased CASK knockout-mediated sorafenib-induced SMMC-7721-sora cell death by CCK8 assay. Cells pretreated with or without transiently transfected with si-NC/si-JNK for 12 h and following treated with 10 μM sorafenib for another 72 h. **(I)** Western blotting were performed to detect the expression of p-JNK and LC3B following the treatment of si-JNK in SMMC-7721-sora sg-CASK cell. **(J)** CASK knockout or control cells transiently transfected with GFP-LC3 plasmid were pretreated with or without 20 μM SP600125 for 2 h. Then, cells were incubated with 12.5 μM sorafenib for 36 h. And the number of GFP-LC3 dots were visualized by confocal microscopy and quantified. *P < 0.05, **P < 0.01, ***P < 0.001.

In the next step, we want to figure out whether CASK depletion-triggered autophagy showed a pro-survival or pro-death role, and whether it is mediated by JNK/c-Jun signaling pathway. Hence, a pan-caspase inhibitor (Z-VAD-FMK) was applied to CASK knockout treatment at first. And the CCK-8 assay indicated that Z-VAD-FMK treatment partially reversed CASK knockout-induced cell death (**Figure 5A**). It suggested that CASK was involved in the regulation of sorafenib resistance by regulating apoptosis, but non-apoptotic form of cell death might exist. Next, an autophagy inhibitor (3-MA) and the siRNA of LC3B were applied to inhibit autophagy. Western blotting was performed to detect the effect of 3-MA and si-LC3B (**Figure S3B**). The CCK-8 assay indicated that 3-MA and si-LC3B treatment noticeably suppressed CASK knockout-induced cell death (**Figures 5B, C**). Then, to determine whether CASK knockout activated autophagic cell death to sensitize HCC cells of sorafenib *in vivo*, xenograft tumor models of SMMC-7721-sora sg-CASK cells were generated. A total of 16 male nude athymic mice were randomly divided into four groups, including the control group, 3-MA group, sorafenib plus control group, and sorafenib plus 3-MA group. As shown in **Figures 5D, E**, the tumor size of 3-MA group was significantly larger than that without 3-MA treated group, especially in combination with sorafenib treated. Taken together, all these data support that inhibition of autophagy attenuates CASK knockout-induced cell death.

To determine whether CASK knockout-induced autophagy was dependent on JNK pathway activation, we specifically inhibited JNK signaling pathway pretreated with JNK-specific inhibitor SP600125, and CCK8 assay showed that inhibition of JNK/c-Jun signaling pathway with SP600125 attenuated the cytotoxicity activity of sorafenib in CASK knockout cells (**Figure 5F**). Western blotting data further showed that SP600125-mediated inhibition of JNK significantly decreased the expression levels of JNK phosphorylation and LC3B-II in CASK knockout cells (**Figure 5G**). Similar consequence was occurred when JNK/c-Jun pathway was inhibited by siRNA of JNK (**Figures 5H, I**). In accordance with these results, laser confocal images indicated that the number of LC3 positive puncta in CASK knockout cells were obviously increased than in control cells, and SP600125 pre-treatment significantly inhibited CASK knockout-induced LC3 positive puncta numbers (**Figure 5J**). In addition, immunohistochemistry analyses of mice tumor tissues also showed that LC3B-II expression, p-JNK expression and p-c-Jun expression were higher in sg-CASK tissues than that in the control tissues with or without sorafenib treated (**Figure S3C**). These data illustrated that JNK/c-Jun signaling pathway was involved in CASK-mediated autophagy.

However, during the therapy, many patients gradually develop resistance to not just one drug, but also to many different drugs. This phenomenon is also called as multidrug resistance and it will seriously affect the therapy efficiency. ATP-binding cassette (ABC) transporters are abundantly expressed in various human tissues including liver and play a crucial role in absorption, distribution, and excretion of drugs, and also be reported to be closely related with drug resistance (29, 30). Besides, we found that suppression of CASK inducing drug-sensitizing effect could be observed in other anticancer drugs, including doxorubicin and daunorubicin (**Figures S4A, B**). By pumping the drugs outside from cancer cells and attenuate the potency of chemotherapeutics, ATP-binding cassette (ABC) transporters superfamily often involves in chemoresistance (31–33). At first, we studied the expression changes of those drug efflux pump proteins closely related with drug resistance in HCC. qRT-PCR indicated that the mRNA expressions of ATP binding cassette subfamily C member 3 (ABCC3) and ABCG2 were significantly decreased when knockdown of CASK (**Figures S4C, D**), and positive correlation were found between CASK and ABCC3 or ABCG2 from GEPIA (http://gepia.cancer-pku.cn/) and starbase (http://starbase.sysu.edu.cn) database (**Figures S4E, F**). While knockout of CASK only significantly downregulated the protein level of ABCG2 when treated with sorafenib, but not MRP3 (**Figures S4G, H**). All results confirmed that ABCG2 might involves in CASK-regulated chemoresistance of HCC cells and more studies should be launched in the future.

## Discussion

Chemoresistance is one of the major obstacles to improve the life quality and survival time of HCC patients. Elucidation the mechanism of drug resistance will help to identify potential and effective therapeutic targets to reverse drug resistance of HCC. This study illustrated that CASK was important for the sorafenib resistance of HCC cells *in vitro* and *in vivo* and further explored the underlying mechanism of CASK in HCC pathogenesis and progression.

Firstly, we found that CASK expression was significantly upregulated in HCC and was closely related with poor prognosis for HCC patients, which was regulated by promoter hypomethylation. More importantly, our data first showed that CASK depletion-mediated sorafenib sensitization *in vitro* and *in vivo* mainly through increasing apoptosis and autophagy. It’s well to known that the cytotoxic effect of chemotherapeutic drugs relies on their ability to induce apoptosis, also known as programmed cell death. Importantly, evading apoptosis is a common and key characteristic of cancer cells and is responsible for chemoresistance (34, 35). In the current study, the experimental data revealed that CASK downregulation increased HCC cell apoptosis through enhancing cleaved caspase 7 activation. Although apoptosis is the most widely studied programmed cell death, recent analyses have highlighted the significance of additional forms of cell death, like autophagic cell death (36–38). In this research, we found that CASK knockout-induced autophagy per se enhanced its cell death effect. Expect apoptosis and autophagy, necrosis is another major mechanism explore for mammalian cell death, and we will demonstrate the relationship between CASK and necrosis in our future study.

Previous researches have indicated that JNK/c-Jun signaling pathway that belongs to mitogen-activated protein kinase (MAPK) pathway, has vital function in regulating autophagic cell death. For example, Bai et al. have reported that PDIA6 knockdown suppressed NSCLC cell proliferation and increased cisplatin-induced autophagic cell death *via* interacting with MAP4K1 to activate the JNK/c-Jun signaling pathway (39); Hu et al. have proved that SNX-2112, the Hsp90 inhibitor, enhanced TRAIL-induced apoptosis and autophagy of cervical cancer cells through activating the ROS-regulated JNK-p53-autophagy-DR5 pathway (26); Zhu et al. indicated that irinotecan (IRI) stimulated the reactive oxygen species (ROS)-related JNK- and p38-MAPK signaling pathways to increase autophagy-dependent apoptosis and inhibit growth of gastric cancer cells (40). Therefore, the active status of the JNK/c-Jun signaling pathway was detected under CASK knockdown condition in the present study. As expected, we indeed observed that phosphorylation of JNK/c-Jun was significantly increased when CASK knockdown with the presence or absence of sorafenib treatment. In addition, inhibition of JNK *via* SP600125 or siRNA markedly suppressed sorafenib induced-autophagy in CASK knockdown SMMC-7721-sora cells. The consequences indicated that the JNK/c-Jun pathway was partially responsible for CASK knockout-medicated autophagic cell death.

An active efflux mechanism is one of the main reasons for multi-drug resistance in cancer. Recently, mounting studies have highlighted the critical role of ABCG2 in mediating multidrug resistance of HCC cells (41, 42). Our study first revealed that ABCG2 might involves in CASK-regulated chemoresistance, providing a novel insight into how CASK regulated sorafenib resistance in HCC. Besides, in order to further improve the clinical value of our experimental study, the clinical significance of CASK detection in sorafenib sensitivity or resistance HCC tissues in predicting the chemotherapy response or survival rate is also worth study in the future.

**Figure 6:**
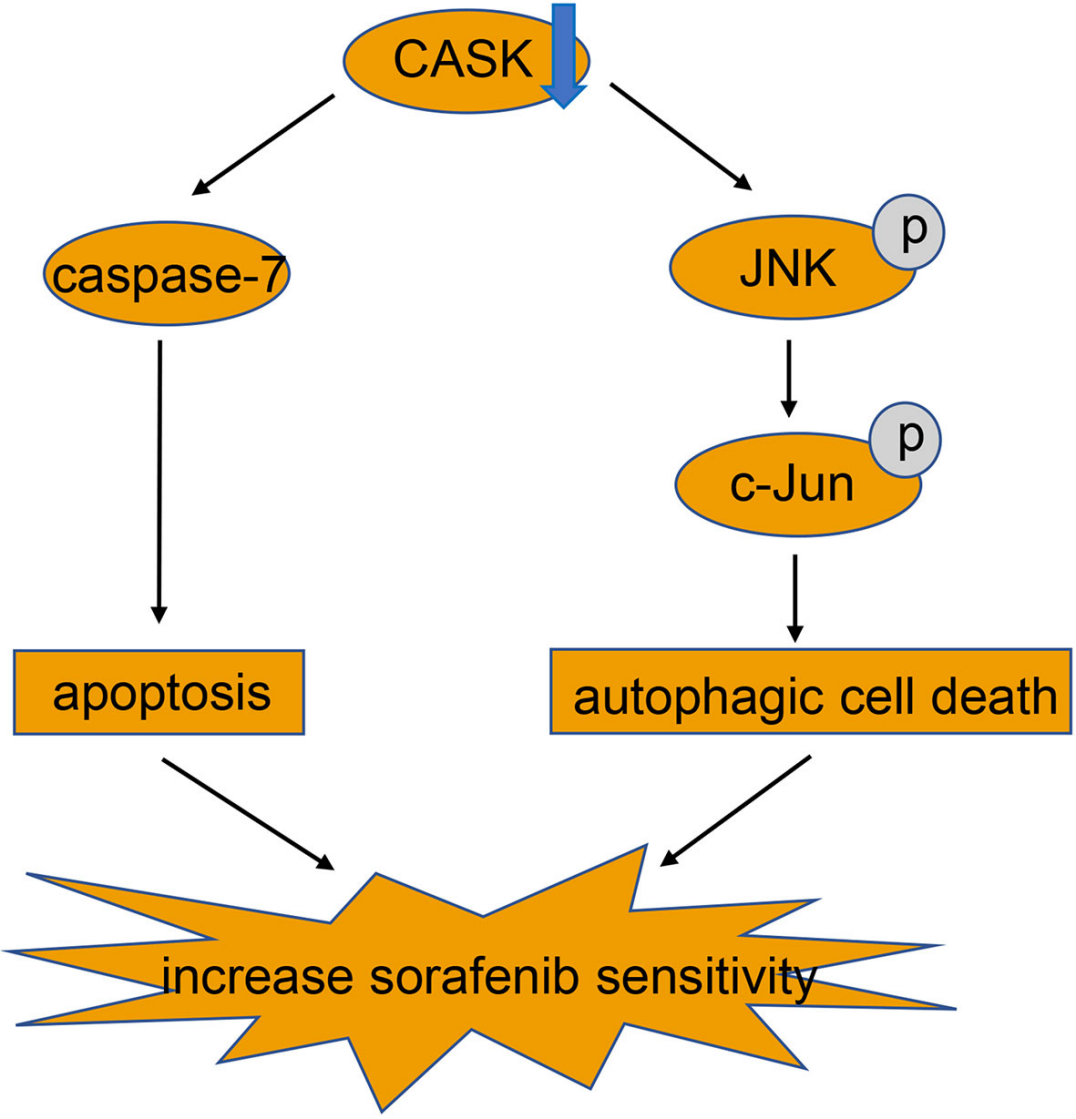
Mechanistic model for CASK regulation of sorafenib-induced apoptosis and autophagy in HCC.

In conclusion, our study demonstrated that hypomethylation-induced upregulation of CASK in HCC is associated with poor prognosis for HCC patients. Furthermore, activation of apoptosis and JNK/c-Jun signaling pathway mediated autophagic cell death produced by CASK downregulation, which reinforces sorafenib’s effect in HCC cells (**Figure 6**). Thus, CASK may serve as a potential novel prognostic indicator in HCC, and targeting CASK may be a promising strategy for HCC patients, especially for sorafenib resistant HCC patients.

## Conclusions

In summary, our analysis for the first time showed that hypomethylation-mediated high expression of CASK in hepatocellular carcinoma is associated with poor prognosis for hepatocellular carcinoma patients, and depletion of CASK enhances the sorafenib sensitivity *in vitro* and *in vivo* through activating apoptosis and autophagic cell death.

## Data Availability Statement

The datasets presented in this study can be found in online repositories. The names of the repository/repositories and accession number(s) can be found in the article/**Supplementary Material**.

## Author Contributions

BD, WF and WL designed the experiments. BD, CB and LJ performed the experiments. BD and LX wrote the manuscript and analyzed the data. Discussion, supervision of all the work and review of this manuscript was done by WL and WF. All authors contributed to the article and approved the submitted version.

## Funding

This research was supported by the National Natural Science Foundation of China (81874225) and 2019 Jiaxing Key Discipiline of Medicine—Oncology (Supporting subject, No. 2019-ZC-11).

## Conflict of Interest

The authors declare that the research was conducted in the absence of any commercial or financial relationships that could be construed as a potential conflict of interest.

## Abbreviations

HCC, hepatocellular carcinoma; CASK, calcium/calmodulin-dependent serine protein kinase; CCK8, cell counting kit-8; 3-MA, 3-Methyladenine; 5-Aza, decitabine; EMT, Epithelial–mesenchymal transition; EGFR, Epidermal growth factor receptor; MAGUK, membrane-associated guanylate kinase; 5-FU, Fluorouracil; ABC, ATP-binding cassette; ABCG2, ATP binding cassette subfamily G member 2; ABCC3, ATP binding cassette subfamily C member 3; TEM, Transmission electron microscopy; DMSO, Dimethyl sulfoxide; DAPI, 4′,6-diamidino-2-phenylindole; H&E, Hematoxylin and eosin; IHC, immunohistochemistry; mRNA, messenger RNA; TCGA, The Cancer Genome Atlas; HPA, human protein atlas; ROC, Receiver operating characteristic; siRNA, small interfering RNA; qRT-PCR, quantitative real-time PCR; GSEA, gene set enrichment analysis; JNK, c-Jun N-terminal kinase; p-JNK, phosphorylated-JNK; p-c-Jun, phosphorylated-c-Jun; MAPK, mitogen-activated protein kinase; ROS, reactive oxygen species.
