# Supplementary material for: CASK Silence Overcomes Sorafenib Resistance of Hepatocellular Carcinoma Through Activating Apoptosis and Autophagic Cell Death
Source: Front Oncol. 2021 Jun 23;11:681683. doi: 10.3389/fonc.2021.681683 (PMC8260832; doi:10.3389/fonc.2021.681683)
Supplement: Supplementary file 1 [file DataSheet_1.pdf]

Supplementary Figure legends:

Figure S1. (a) The mRNA expression of CASK in 3 HCC cell lines (SMMC-7721, SMMC-7721-sora, SK-Hep-1) were compared with 1 normal liver cell line (QSG7701). (b-c) The mRNA expression of CASK in a dose-dependent manner in the presence of sorafenib. (d-f) The effect of si-CASK in SMMC-7721-sora, SMMC-7721 and SK-Hep-1 cells were detected by performing qRT-PCR analysis. (g) The effect of si-CASK-3 in SMMC-7721-sora, SMMC-7721 and SK-Hep-1 cells were detected by performing western blotting. **$P<0.01$, ***$P<0.001$, ****$P<0.0001$.

Figure S2. (a) The effect of pcDNA-CASK in SMMC-7721-sora, SMMC-7721 and SK-Hep-1 cells were detected by performing western blotting. (b) CCK-8 assay analysis of the impact of CASK overexpression combine with IC50 concentrations of sorafenib on SMMC-7721-sora, SMMC-7721 and SK-Hep-1 cells growth. (c, d) Colony information assay showing the effects of CASK overexpression on SMMC-7721-sora, SMMC-7721 and SK-Hep-1 cells growth with sorafenib treated. (e, f) The apoptosis rate in CASK overexpression or control cells with sorafenib treated were measured by flow cytometry analysis. *$P<0.05$.

Figure S3. (a) Western blot analysis showing levels of autophagy-related proteins in CASK overexpression cells with the presence of sorafenib (sorafenib treated concentration: SMMC-7721: 10μM, SMMC-7721-sora: 20μM, SK-Hep-1: 15μM; treated time: 72h). (b) Western blot analysis showing the effect of 3-MA and si-LC3B. (c) Immunohistochemical staining (IHC) of tumor samples were performed to analyze CASK, LC3B, p-JNK and p-c-Jun expression. Scale bar: 100 μM.

Figure S4. (a, b) The sensitivities of doxorubicin and daunorubicin in HCC cells were increased by CASK knockout. (c, d) mRNA expressions of ABC transporters were measured in SMMC-7721-sora and SMMC-7721 cells that transiently transfected with si-NC or si-CASK-3. (e, f) Correlation analysis between CASK and ABCC3, ABCG2 from GEPIA (http://gepia.cancer-pku.cn/) and starbase (http://starbase.sysu.edu.cn)

database. (g, h) Western blotting analysis of ABCG2 and MRP3 protein levels in CASK knockout or control cells, with or without sorafenib treated (sorafenib treated concentration: SMMC-7721: 10μM, SMMC-7721-sora: 20μM; treated time: 72h). **P<0.01, ****P<0.0001.

Supplementary Figure:

Figure S1:

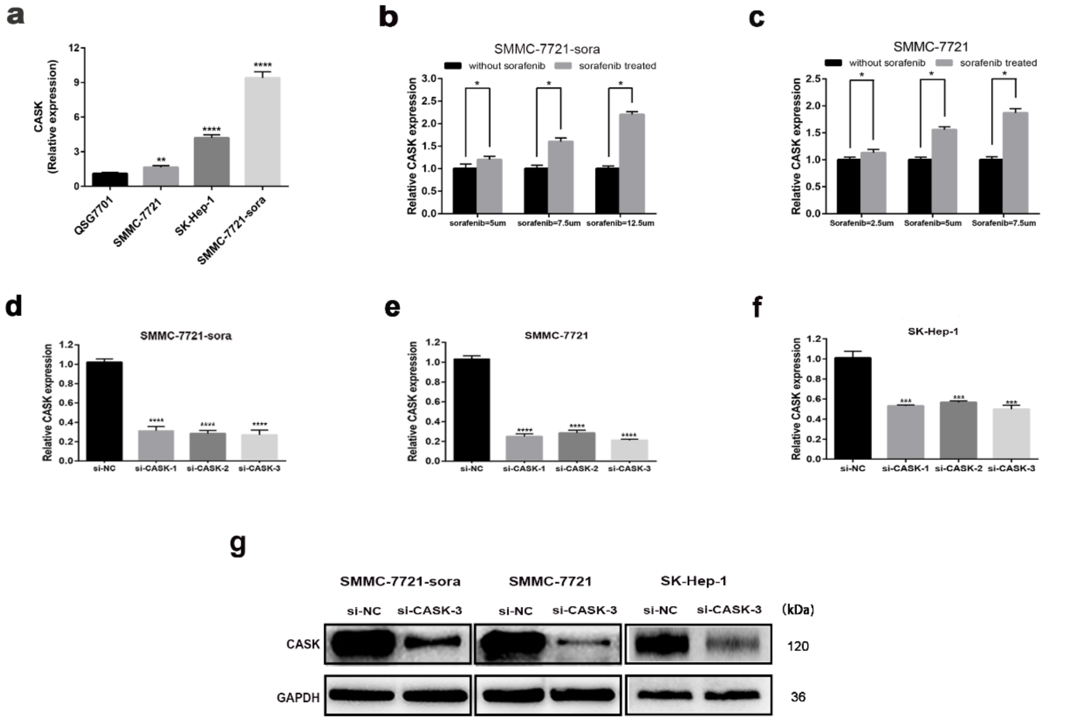

Figure S2:

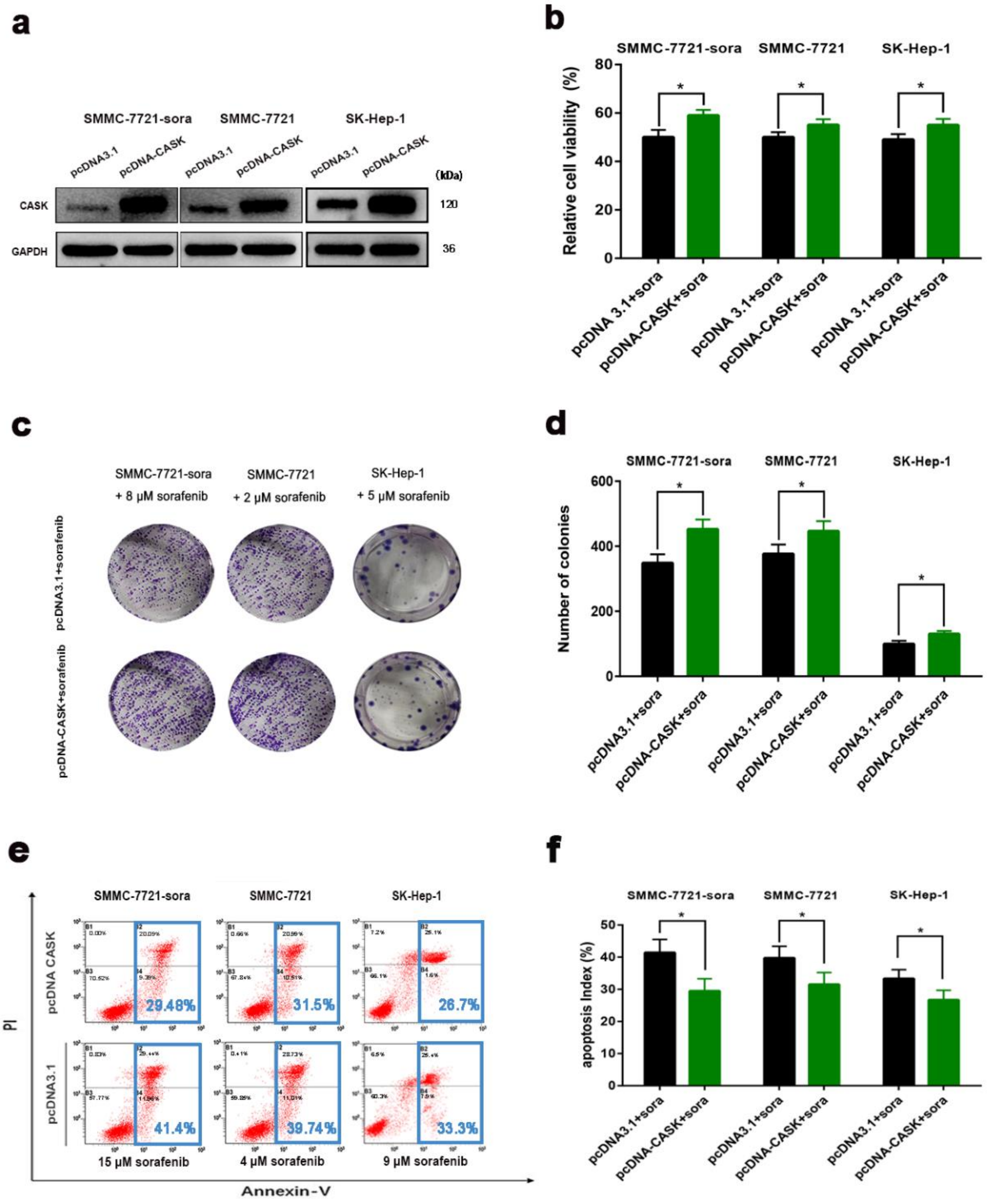
a
SMMC-7721-sora
SMMC-7721
SK-Hep-1
pcDNA3.1
pcDNA-CASK
(kDa)
CASK
120
GAPDH
36
b
SMMC-7721-sora
SMMC-7721
SK-Hep-1
Relative cell viability (%)
pcDNA 3.1+sora
pcDNA-CASK+sora
c
SMMC-7721-sora + 8 μM sorafenib
SMMC-7721 + 2 μM sorafenib
SK-Hep-1 + 5 μM sorafenib
pcDNA3.1+sorafenib
pcDNA-CASK+sorafenib
d
Number of colonies
pcDNA3.1+sora
pcDNA-CASK+sora
e
pcDNA CASK
pcDNA3.1
PI
29.48%
31.5%
26.7%
41.4%
39.74%
33.3%
15 μM sorafenib
4 μM sorafenib
9 μM sorafenib
Annexin-V
f
apoptosis Index (%)

Figure S3:

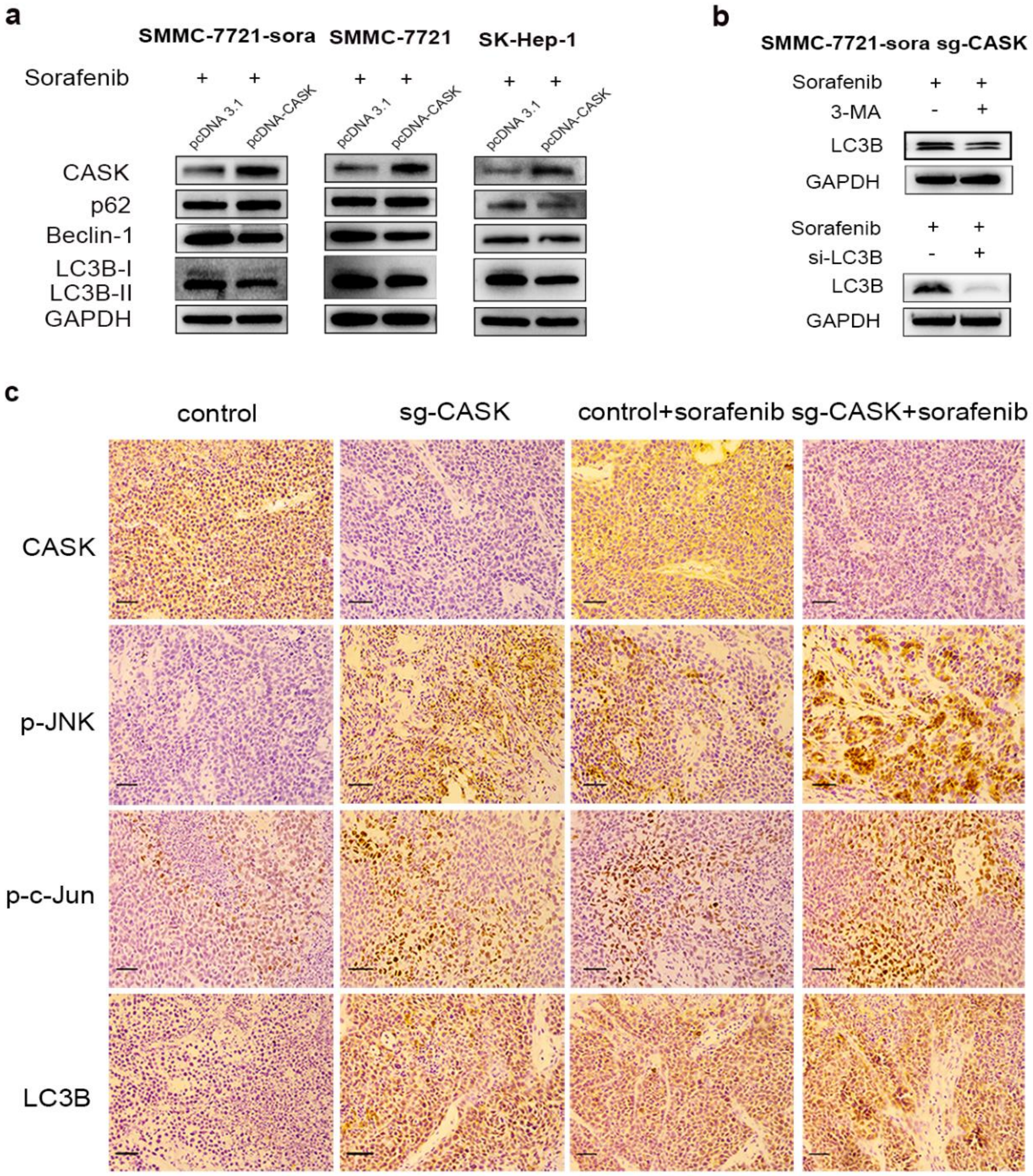
a
SMMC-7721-sora
SMMC-7721
SK-Hep-1
Sorafenib
+ + + + + +
pcDNA 3.1
pcDNA-CASK
CASK
p62
Beclin-1
LC3B-I
LC3B-II
GAPDH
b
SMMC-7721-sora sg-CASK
Sorafenib + +
3-MA - +
LC3B
GAPDH
Sorafenib + +
si-LC3B - +
LC3B
GAPDH
c
control
sg-CASK
control+sorafenib
sg-CASK+sorafenib
CASK
p-JNK
p-c-Jun
LC3B

Figure S4:

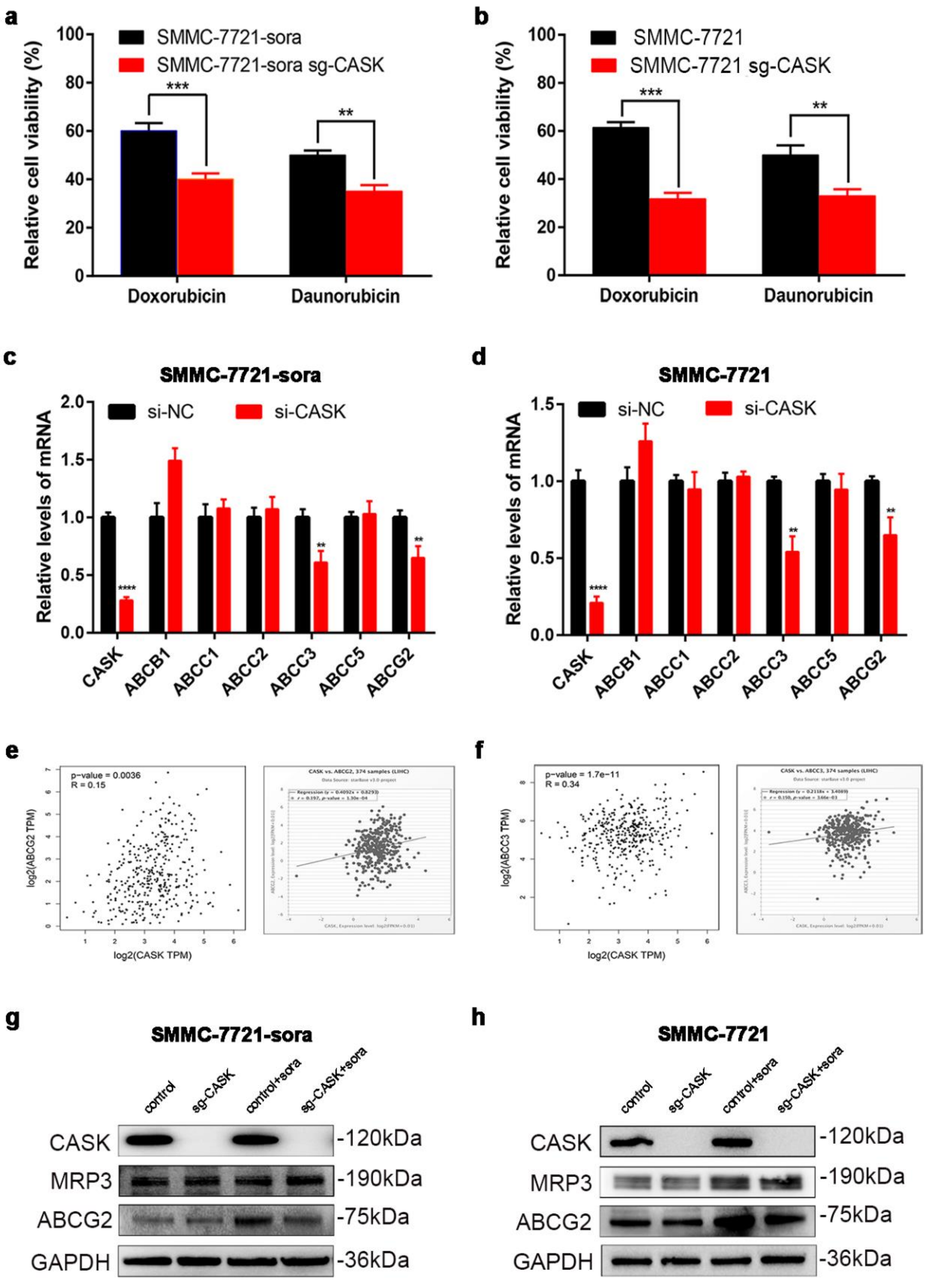
a
SMMC-7721-sora
SMMC-7721-sora sg-CASK
Relative cell viability (%)
100
80
60
40
20
0
***
**
Doxorubicin
Daunorubicin
b
SMMC-7721
SMMC-7721 sg-CASK
Relative cell viability (%)
***
**
Doxorubicin
Daunorubicin
c
SMMC-7721-sora
si-NC
si-CASK
Relative levels of mRNA
2.0
1.5
1.0
0.5
0.0
****
**
**
CASK
ABCB1
ABCC1
ABCC2
ABCC3
ABCC5
ABCG2
d
SMMC-7721
si-NC
si-CASK
Relative levels of mRNA
1.5
1.0
0.5
0.0
****
**
**
CASK
ABCB1
ABCC1
ABCC2
ABCC3
ABCC5
ABCG2
e
p-value = 0.0036
R = 0.15
log2(ABCG2 TPM)
log2(CASK TPM)
f
p-value = 1.7e-11
R = 0.34
log2(ABCC3 TPM)
log2(CASK TPM)
g
SMMC-7721-sora
control
sg-CASK
control+sora
sg-CASK+sora
CASK
MRP3
ABCG2
GAPDH
-120kDa
-190kDa
-75kDa
-36kDa
h
SMMC-7721
control
sg-CASK
control+sora
sg-CASK+sora
CASK
MRP3
ABCG2
GAPDH
-120kDa
-190kDa
-75kDa
-36kDa

Supplementary Table S1: Gene primers and siRNA consequence.

Gene	List	Target sequence
CASK	gene primer	FP: TGGAAGCTCTACGCTACTGC
		RP: GTTTAACAGGTGCCGAGTTTTC
	si-CASK-1	5’-GAATAACGACGCAAAGGAA-3’
	si-CASK-2	5’-GAGCACAATTTGAATATGA-3’
	si-CASK-3	5’-GCUGAAAGGAUCACUGUUUtt-3’
ABCB1	gene primer	FP: TTGCTGCTTACATTCAGGTTTCA
		RP: AGCCTATCTCCTGTCGCATTA
ABCC1	gene primer	FP: CTCTATCTCTCCCGACATGACC
		RP: AGCAGACGATCCACAGCAAAA
ABCC2	gene primer	FP: TCTCTCGATACTCTGTGGCAC
		RP: CTGGAATCCGTAGGAGATGAAGA
ABCC3	gene primer	FP: CACCAACTCAGTCAAACGTGC
		RP: GCAAGACCATGAAAGCGACTC
ABCC5	gene primer	FP: GAACTCGACCGTTGGAATGC
		RP: TCATCCAGGATTCTGAGCTGAG
ABCG2	gene primer	FP: ACGAACGGATTAACAGGGTCA
		RP: CTCCAGACACACCACGGAT
LC3B	si-LC3B	5'-CACCUUCGAACAAAGAGUAdTdT- 3'
JNK	si-JNK	5'-GUUCCCAGGUACAGAUCAUTT- 3'
GAPDH	gene primer	FP: CTGGGCTACACTGAGCACC
		RP: AAGTGGTCGTTGAGGGCAATG

Supplementary Table S2: primary antibodies.

Antibody	Concentration for WB	Specificity	Company	Product id
GAPDH	1:10000	Rabbit	abcam	ab181602
CASK	1:1000	Mouse	abcam	ab252540
Caspase-7	1:1000	Rabbit	CST	12827
LC3B	1:1000	Rabbit	CST	3868
SQSTM1/p62	1:1000	Rabbit	CST	8025
Beclin-1	1:2000	Rabbit	abcam	ab207612
JNK	1:1000	Rabbit	abcam	ab179461
p-JNK	1:1000	Rabbit	CST	4668
c-Jun	1:1000	Rabbit	abcam	ab40766
p-c-Jun	1:1000	Rabbit	abcam	ab32385
MRP3	1:20	Mouse	abcam	ab3375
ABCG2	1:1000	Rabbit	abcam	ab207732
